# The Oligosaccharyltransferase Catalytic Subunit PsSTT3B Is Required for Asexual Development and Pathogenicity in *Phytophthora sojae*

**DOI:** 10.3390/jof12040274

**Published:** 2026-04-09

**Authors:** Quanhe Ma, Borui Zhang, Tongshan Cui, Shanshan Chen, Shan Geng, Fan Zhang, Can Zhang, Xili Liu

**Affiliations:** 1 College of Plant Protection, China Agricultural University, Beijing 100193, China; maqh191@163.com (Q.M.); zhangbr96@163.com (B.Z.); cuitongshan0619@163.com (T.C.); 18800172207@163.com (S.C.); 13223262791@163.com (S.G.); fan_zhang@cau.edu.cn (F.Z.); czhang@cau.edu.cn (C.Z.); 2 School of Life Sciences, Tsinghua University, Beijing 100084, China; 3 State Key Laboratory of Crop Stress Biology for Arid Areas, College of Plant Protection, Northwest A&F University, Yangling 712100, China

**Keywords:** *Phytophthora sojae*, PsSTT3B, oligosaccharyltransferase, development, virulence

## Abstract

*N*-glycosylation is a fundamental post-translational modification that contributes to protein folding, stability, and secretion in eukaryotes. The catalytic subunit STT3 of the oligosaccharyltransferase complex mediates the transfer of preassembled oligosaccharides to nascent polypeptides in the endoplasmic reticulum. Here, we identified and functionally characterized *PsSTT3B*, one of the *STT3* paralogs in *Phytophthora sojae* (*P. sojae*). *PsSTT3B* plays an important role in the growth, development, and pathogenicity of *P. sojae.* CRISPR/Cas9-mediated deletion of *PsSTT3B* resulted in reduced vegetative growth, sporangia production, and zoospore production in *P. sojae*. *PsSTT3B* deletion mutants demonstrated significantly reduced virulence on soybean leaves and etiolated seedlings. Importantly, *PsSTT3B* deletion mutants also exhibited reduced zoospore germination and diminished chemotaxis toward soybean isoflavones. Moreover, deletion of *PsSTT3B* increased sensitivity to tunicamycin and dithiothreitol and influenced the ConA-binding glycoprotein profile. Our findings show that *PsSTT3B* is associated with asexual development, virulence, and sensitivity to ER stress-related conditions of *P. sojae*. Our study suggests that *PsSTT3B* represents a potential candidate gene for the prevention and control of *P. sojae*.

## 1. Introduction

Protein glycosylation represents one of the most abundant and evolutionarily conserved post-translational modifications in eukaryotic cells [1]. It has been estimated that more than half of eukaryotic proteins are glycoproteins, among which approximately 90% carry N-glycans [2]. During this process, a preassembled lipid-linked oligosaccharide (LLO; Glc_3_Man_9_GlcNAc_2_) is transferred en bloc from the lipid carrier dolichol pyrophosphate to an asparagine residue within the consensus sequon N-X-S/T (X ≠ Pro) of a nascent polypeptide [3]. *N*-glycosylation occurs primarily co-translationally within the lumen of the endoplasmic reticulum (ER), although post-translational modification can also occur, and it profoundly influences protein folding, quality control, stability, trafficking, secretion, and enzymatic activity [1,4].

The transfer of oligosaccharide moieties is catalyzed by the oligosaccharyltransferase (OST) complex, a highly conserved enzyme complex resident in the ER. The composition and diversity of the OST complex vary across eukaryotic lineages. In most eukaryotes, the OST complex comprises multiple subunits, with STT3 serving as the catalytic core [5]. However, in some protists, multiple STT3 paralogs are present, whereas several non-catalytic OST subunits are absent, resulting in single-subunit OST (ssOST) enzymes that can exhibit distinct substrate specificities [6,7,8,9]. Together, these observations support the view that STT3-containing OST systems have diversified during eukaryotic evolution, with variation in subunit composition and substrate selectivity.

STT3 also exhibits isoform diversification in many eukaryotes. In animals and plants, two STT3 isoforms (STT3A and STT3B) perform partially overlapping but distinct functions in *N*-glycosylation [10]. STT3A primarily associates with the Sec61 translocon–ribosome complex to mediate co-translational glycosylation, whereas STT3B predominantly supports post-translational glycosylation and can modify sequons skipped during co-translation [11,12,13]. This division of labor broadens glycosylation coverage and facilitates efficient maturation of nascent glycoproteins. Loss of either STT3A or STT3B can severely affect growth and viability, depending on the organism and genetic context [14,15,16,17,18]. In fungi, STT3 is typically encoded by a single essential gene, and its repression or deletion results in severe growth defects, impaired glycoprotein secretion, and reduced virulence [19,20,21].

In contrast to these characterized systems, the roles of STT3 homologs in oomycetes remain largely unexplored. Oomycetes are filamentous eukaryotic microorganisms within the Stramenopile lineage and comprise numerous economically important pathogens of crops and forest species [22]. Among these pathogens, *Phytophthora sojae* (*P. sojae*) is a soilborne oomycete that causes root and stem rot in soybean and is responsible for substantial economic losses worldwide [23,24]. Despite extensive investigations into effector biology and host–pathogen interactions in *P. sojae*, the role of *PsSTT3*-mediated *N*-glycosylation in its development and virulence remains poorly understood.

A recent comparative study reported that most species in the genera *Phytophthora* and *Pythium* contain two STT3 genes, STT3A and STT3B, which diverged prior to genus separation [25]. In *P. capsici*, deletion of *PcSTT3B* impaired vegetative growth, development, glycoprotein content, and virulence. Deletion of *PcSTT3B* also increased sensitivity to tunicamycin and induced compensatory upregulation of *PcSTT3A* transcripts, indicating partial functional redundancy [25]. In parallel, recent work on *PsSTT3A* demonstrated that homozygous deletion of *PsSTT3A* is lethal, underscoring its essential role. Partial silencing of *PsSTT3A* reduced vegetative growth, sporulation, zoospore production, glycoprotein biogenesis, and virulence [26]. These findings establish that STT3-mediated glycosylation plays a pivotal role in the development and pathogenicity of *P. sojae*.

However, the functional role of *PsSTT3B* in *P. sojae* remains unknown. Given the partial redundancy and specialization observed in other systems [27,28], it is unclear whether *PsSTT3B* performs overlapping or distinct functions relative to *PsSTT3A*. In particular, whether *PsSTT3B* contributes to asexual development, zoospore functionality, chemotaxis, ER stress tolerance, and virulence in soybean has not been systematically examined.

Therefore, this study aimed to systematically elucidate the functional roles of *PsSTT3B* in the growth, asexual development, and pathogenicity of *P. sojae* and to evaluate its involvement in ER stress responses. We identified and experimentally validated the *PsSTT3B* gene in *P. sojae* and investigated its biological function through CRISPR/Cas9-mediated gene deletion and complementation. The CRISPR–Cas9 system offers high targeting specificity and efficient gene knockout, enabling reliable validation of loss-of-function phenotypes and thereby providing a strong rationale for its application in this study. We show that *PsSTT3B* is required for normal vegetative growth, sporangia production, zoospore production, germination, chemotactic responses, and full virulence. Furthermore, deletion of *PsSTT3B* is associated with changes in the ConA-binding glycoprotein profile and increased sensitivity to ER stress-related conditions, suggesting that *PsSTT3B* may influence asexual development, pathogenicity, and ER homeostasis in *P. sojae*.

## 2. Materials and Methods

### 2.1. Study Materials

The wild-type (WT) strain P6497 was used for transformation and gene-editing experiments. P6497 and its transformants were routinely cultured on 10% *v*/*v* V8 agar at 25 °C in the dark [29]. The susceptible soybean cultivar Williams was grown in a greenhouse at 27 ± 2 °C, 80% relative humidity, and a 16-h photoperiod. For etiolated seedlings, cv. Williams plants were grown in plastic pots containing vermiculite at 25 °C in the dark for 7–9 days.

### 2.2. Identification of the P. sojae STT3B Gene

The amino acid sequence of PsSTT3B was used as a query to perform a tBLASTn (https://blast.ncbi.nlm.nih.gov/Blast.cgi?PROGRAM=tblastn&PAGE_TYPE=BlastSearch&LINK_LOC=blasthome, accessed on 6 September 2023) search against the two high-quality *P. sojae* genome assemblies, P6497_PHYSO3 (AAQY00000000.2) and P6497_smrtdenovo_ONT10kb_corrected (WWEI01000000.1), deposited in NCBI. The identified PsSTT3B sequence was amplified from the *P. sojae* isolate P6497 using gene-specific primers designed with Primer3 (https://bioinfo.ut.ee/primer3-0.4.0/, accessed on 8 September 2023) and listed in Table S1.

STT3 protein sequences from representative species were retrieved from the National Center for Biotechnology Information (NCBI; https://static.pubmed.gov/portal/portal.fcgi/, accessed on 6 September 2023) and FungiDB databases (https://fungidb.org/fungidb/app, accessed on 6 September 2023) [30]. Multiple sequence alignment was performed, and a phylogenetic tree was constructed using the neighbor-joining (NJ) method in MEGA 7.0 with 1000 bootstrap replicates [31]. Conserved motifs were identified using the MEME suite (https://meme-suite.org/meme/, accessed on 3 March 2026). Transmembrane domains of PsSTT3B were predicted using the TMHMM Server (https://services.healthtech.dtu.dk/services/TMHMM-2.0/, accessed on 3 March 2026).

### 2.3. Vector Construction

*PsSTT3B* deletion transformants were generated using the CRISPR/Cas9 system [32,33]. The sgRNA for CRISPR/Cas9-mediated gene deletion was designed using the EuPaGDT server (http://grna.ctegd.uga.edu, accessed on 30 September 2023) and cloned into the pYF515-sgRNA-Cas9 vector following a previously described protocol. The resulting plasmid was named pYF515-sgRNA-Cas9-STT3B. The 1 kb upstream and 1 kb downstream sequences of the *PsSTT3B* gene were amplified and cloned into the pBSK+ donor vector, and the replacement gene *GFP* was inserted between them. The resulting plasmid was named pBSK+-*STT3B*. A total of 40–50 µg of plasmid DNA (pYF515-sgRNA-Cas9-*STT3B*, pBSK+-*STT3B*) was used for co-transformation to delete the *PsSTT3B* gene. The entire *PsSTT3B* gene, ligated with its two flanking 1 kb fragments, was cloned into the pBSK+ donor vector and named pBSK+-*STT3B*-C. The plasmids pYF515-PcMuORP1-N (targeting *GFP*), pBSK+-STT3B-C, and pYF-Cas9-EI were co-transformed for gene complementation [34]. The primers and sgRNA sequences used in this study are listed in Table S1.

### 2.4. P. sojae Transformation

Gene disruption and in situ complementation were performed using a CRISPR/Cas9-mediated gene replacement strategy [32,33,34]. Briefly, 3-day-old *P. sojae* mycelial mats cultured in liquid pea broth were rinsed with ultrapure water and suspended in 0.8 M mannitol with gentle shaking. After incubation at 25 °C for 10 min, the mycelium was transferred to 20 mL of enzyme solution (0.4 M mannitol, Sigma, St. Louis, MO, USA; 20 mM KCl, Sigma, St. Louis, MO, USA; 20 mM 2-(N-morpholino)ethanesulfonic acid (MES), Sigma, St. Louis, MO, USA; 10 mM of CaCl_2_, Sigma, St. Louis, MO, USA; 0.3 g of lysing enzymes from *Trichoderma harzianum*, Sigma, St. Louis, MO, USA; 0.3 g of Cellulysin^®^ cellulase, Calbiochem, San Diego, CA, USA; pH 5.7) and incubated at 25 °C for approximately 40 min with gentle shaking (55–60 rpm).

The mixture was filtered through two layers of Miracloth (EMD Millipore Corp., Billerica, MA, USA), and the resulting protoplasts were collected via centrifugation at 530× *g* for 4 min. After washing with 30 mL of W5 solution (5 mM KCl; 125 mM CaCl_2_; 154 mM NaCl; Sigma, St. Louis, MO, USA; and 173 mM glucose, Sigma, St. Louis, MO, USA), the protoplasts were resuspended in 5–10 mL of W5 solution and incubated on ice for 30 min. The protoplasts were precipitated again via centrifugation at 530× *g* for 5 min and resuspended at a concentration of 1 × 10^6^–1 × 10^7^ protoplasts mL^−1^ in MMg solution (0.4 M mannitol; 15 mM MgCl_2_, Sigma, St. Louis, MO, USA; and 4 mM MES; pH 5.7).

Co-transformation was performed using 1 mL of protoplast suspension with 40–50 µg of each plasmid. The protoplast–plasmid mixtures were incubated on ice for 20 min, followed by the gradual addition of 1740 µL of freshly prepared PEG solution (40% (*m*/*v*) polyethylene glycol (PEG) 4000, Sigma, St. Louis, MO, USA; 0.2 M mannitol; and 0.1 M CaCl_2_). The mixtures were gently shaken and incubated on ice for an additional 20 min before 20 mL of pea broth (0.5 M mannitol; 9 mM CaCl_2_; and 20 mM CaCO_3_, Sigma, St. Louis, MO, USA) was added. The samples were then incubated in the dark at 18 °C for 24 h to allow regeneration.

Transformants were selected on V8 agar supplemented with 50 µg mL^−1^ G418 or 0.01 µg mL^−1^ oxathiapiprolin (DuPont Crop Protection, Wilmington, DE, USA) [32,33,34]. PCR was performed to verify the replacement of *PsSTT3B* with *GFP* in deletion mutants, and the amplified fragments were further confirmed by Sanger sequencing. *PsSTT3B* deletion and complementation transformants were further confirmed by RT-qPCR. A transformation control that retained the native *PsSTT3B* locus was used as the control (CK). The primers used in this study are listed in Table S1.

### 2.5. Transcription Profile of the PsSTT3A and PsSTT3B Genes

Biological samples of *P. sojae* at different developmental stages were collected as previously described [35], including mycelium grown on V8 agar, sporangia, zoospores, germinated cysts, and infected soybean leaves collected at 0, 3, 6, 12, 24, 48, and 72 h post-inoculation (hpi).

Total RNA was extracted from the above samples using the SV Total RNA Isolation Kit (Promega, Beijing, China). RNA integrity was assessed via agarose gel electrophoresis. After DNase I treatment, 1 µg of total RNA was used for cDNA synthesis using the PrimeScript™ RT Reagent Kit (Takara, Beijing, China) according to the manufacturer’s instructions.

RT-qPCR was performed using a qTower 2.2 system with qPCRsoft 3.4 software (Analytik Jena AG, Jena, Germany) and the SYBR Premix Dimer Eraser Kit (CW Biotech, Beijing, China). Cycling conditions were as follows: 95 °C for 2 min, followed by 40 cycles of 95 °C for 10 s and 60 °C for 30 s. *RPS* and *RPL13A* were used as internal reference genes for normalization [26], and relative transcript levels were calculated using the 2^−ΔΔCt^ method [18]. Each sample was analyzed using three independent biological replicates, each comprising three technical replicates. The primers used in this study are listed in Table S1.

### 2.6. Phenotypic Characterization of PsSTT3B Deletion Mutants

To examine mycelial growth, mycelial plugs (5 mm in diameter) were transferred onto V8 agar plates and incubated at 25 °C in the dark for 5 d, with three biological replicates per treatment. Colony diameter was determined by measuring each colony along two perpendicular axes and averaging the values [36,37]. For cellophane overlay assays, sterile, uncoated cellophane membranes (Beijing Tiangong Creation Biotechnology Co., Ltd., Beijing, China) were placed on the surface of V8 agar plates prior to inoculation. Mycelial plugs were then placed onto the membranes and incubated under standard conditions. This approach allows mycelial growth on a detachable surface, facilitating the assessment of surface-associated growth characteristics without agar interference.

To analyze sporangia production, ten mycelial plugs (5 mm in diameter) excised from the margins of actively growing colonies were transferred into Petri dishes containing 20 mL of 10% clarified V8 broth and incubated at 25 °C in the dark for 3 days. The mycelium was then washed five times with 20 mL of sterile distilled water (SDW), after which the Petri dishes were returned to dark incubation at 25 °C for a further 4–8 h to allow sporangia development. The number of sporangia was counted under a light microscope at ×100 magnification [37].

Zoospore production was assessed as described above. After washing the mycelium five times, an additional 10 mL of SDW was added to each Petri dish, and the cultures were incubated in the dark at 25 °C for 8–10 h. Zoospore concentration was subsequently determined using a hemocytometer [36,37].

For post-encystment germination assays, zoospore suspensions were vortexed for 1 min to induce encystment and incubated at 25 °C in the dark for 6 h. More than 100 cysts were examined under a microscope, with a cyst considered germinated when the germ tube length exceeded the cystospore diameter. Germ tube length was likewise measured for at least 100 cysts per replicate [36,37].

To evaluate pathogenicity, the first true leaves of 9-day-old soybean seedlings (cv. Williams) were detached and placed in Petri dishes lined with moist filter paper to prevent dehydration. Ten leaves were used for each treatment. A single mycelial plug was placed at the center of each leaf, and the inoculated leaves were incubated at 25 °C for 4 days. Lesion areas were quantified using Image J (1.54 g). The etiolated seedlings of cv. Williams were inoculated on the hypocotyls with mycelium plugs (5 mm in diameter) and maintained at 25 °C and 80% relative humidity in the dark for 3 days. The lesion lengths were measured. Each strain was tested using six seedlings. All experiments were performed with three independent biological replicates.

### 2.7. Zoospore Chemotaxis Assays of P. sojae

*P. sojae* zoospores were obtained as described above. Capillary tubes (0.9–1.1 mm inner diameter) were filled with 10 nmol L^−1^ isoflavone solution, immediately flame-sealed, and used as chemoattractant sources. Capillary tubes containing an equal volume of SDW served as negative controls [38].

The zoospore suspension was adjusted to a final concentration of 1 × 10^5^ zoospores mL^−1^. A 500 µL aliquot was gently pipetted onto a glass slide, into which the prepared capillary tube was inserted. After 3 min of incubation, zoospore chemotactic behavior was observed and recorded under a light microscope [38].

### 2.8. Sensitivity Assays of P. sojae to TM and DTT

Test strains were cultured on V8 agar at 25 °C in darkness for 3 days. A mycelial plug 5 mm in diameter was excised from the colony periphery using a cork borer and placed mycelium-side down onto V8 agar supplemented with either 0.5 µg/mL TM or 6 mM DTT. Plates were then incubated at 25 °C in darkness for a further 5 days [39].

Colony diameter was determined by measuring each colony along two perpendicular axes and averaging the values. Growth inhibition was calculated as follows:

$$Inhibition(\%)=\frac{Dcontrol-Dtreatment}{Dcontrol-5}\times 100$$

where Dcontrol is the colony diameter on V8 agar without TM or DTT, and Dtreatment is the colony diameter on V8 agar supplemented with TM or DTT.

All experiments were performed with three independent biological replicates.

### 2.9. Statistical Analysis

All experiments were performed with at least three independent biological replicates, each consisting of samples derived from separate cultures or plant materials. Data are presented as means ± standard deviation (SD). Statistical analyses were conducted using one-way analysis of variance (ANOVA) followed by Tukey’s multiple comparison test. Where applicable, data were tested for normality and homogeneity of variance prior to analysis. Significant differences are indicated by asterisks (*, *p* < 0.05; **, *p* < 0.01) or by different letters (*p* < 0.05).

## 3. Results

### 3.1. Identification and Evolutionary Characterization of PsSTT3B in P. sojae

**Figure 1:**
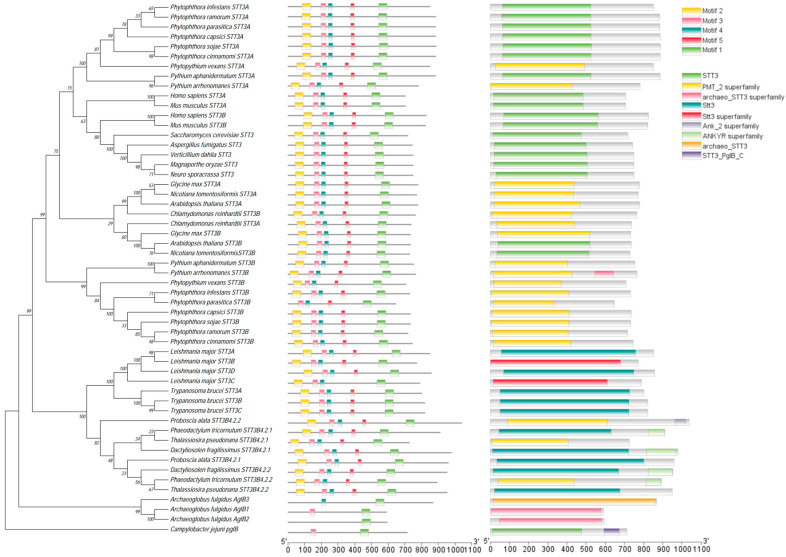
Sequence analysis of the PsSTT3B protein: Neighbor-joining phylogenetic tree of STT3 proteins from oomycetes and other representative taxa. Bootstrap values (%, *n* = 1000 replicates) indicate the support for each node. The scale bar represents 0.20 substitutions per site (i.e., 20% sequence divergence). Branch lengths reflect the evolutionary distances among STT3 proteins. Conserved motifs and domain architectures of STT3 proteins are compared between oomycetes and fungi.

In a previous study, analysis of the *P. sojae* genome identified two paralogous STT3 homologs, designated PsSTT3A and PsSTT3B [26]. Sanger sequencing of genomic DNA and cDNA confirmed that *PsSTT3B* contains a 2202 bp open reading frame encoding a predicted 733-amino-acid protein, thereby validating the gene model (Supplementary Figure S2). Domain prediction analysis revealed that both PsSTT3A and PsSTT3B harbor the conserved STT3/PMT_2 superfamily domain characteristic of catalytic OST subunits (Supplementary Figure S2). Multiple sequence alignment revealed that PsSTT3B contains the highly conserved WWDYG motif, which is essential for catalytic activity and substrate recognition during *N*-glycosylation. In addition, a conserved DXXK motif was identified, consistent with its predicted role in stabilizing substrate interactions during glycan transfer (Figure 1).

Phylogenetic analysis using representative STT3 homologs from oomycetes, fungi, and plants showed that oomycetes possess two distinct STT3 clades corresponding to STT3A and STT3B paralogs (Figure 1). PsSTT3B clustered within the oomycete STT3B clade, indicating strong evolutionary conservation among related species (Figure 1). By contrast, most fungal species contain only a single STT3 homolog (Figure 1).

Protter analysis predicted twelve transmembrane helices in PsSTT3B, consistent with the typical topology of STT3 family proteins (Supplementary Figure S1). These features support that PsSTT3B is an integral membrane protein and are consistent with its predicted association with the endomembrane system, including the ER, where OST-mediated *N*-glycosylation occurs.

Protter analysis further predicted six potential *N*-glycosylation sites within PsSTT3B (N128, N526, N570, N577, N581, and N660) (Supplementary Figures S1 and S2), suggesting that the subunit itself may be *N*-glycosylated. Comparative sequence analysis revealed that three sites (N570, N577, and N581) are conserved in PsSTT3A [26]. Notably, the conservation of these three putative *N*-glycosylation sites suggests potential functional relevance.

### 3.2. Transcriptional Profiling of PsSTT3A and PsSTT3B Across Development and Infection

**Figure 2:**
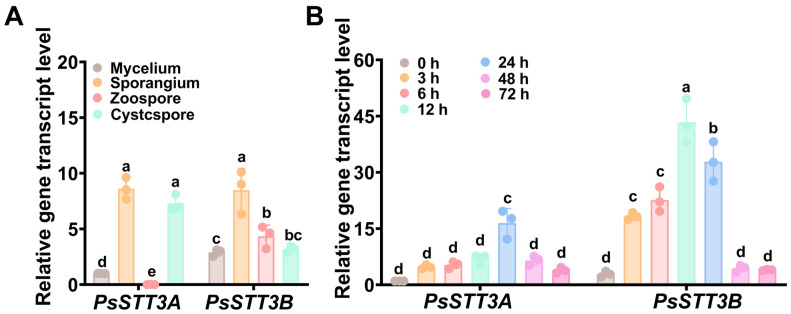
Transcriptional profiling of *PsSTT3A* and *PsSTT3B* across development and infection: (**A**) Relative transcript levels of *PsSTT3A* and *PsSTT3B* at the developmental stages. (**B**) Relative transcript levels of *PsSTT3A* and *PsSTT3B* during infection, measured at 0, 3, 6, 12, 24, 48, and 72 h post-inoculation (hpi). All experiments were repeated three times. Different letters above bars indicate significant differences at *p* < 0.05, as determined by one-way ANOVA with Tukey’s HSD test. Error bars represent the mean ± SD of three biological replicates.

Transcript analysis via RT–PCR across multiple developmental stages of *P. sojae* (including mycelium, sporangia, zoospores, cysts, and germinating cysts) revealed distinct expression patterns for *PsSTT3B* and *PsSTT3A*. Notably, *PsSTT3B* transcript levels were significantly elevated in sporangia compared with mycelium (Figure 2A), suggesting that *PsSTT3B*-mediated *N*-glycosylation may play a particularly important role during the zoospore stage. By contrast, *PsSTT3A* was expressed during vegetative growth and sporangial development; however, its transcript levels were nearly undetectable during the zoospore stage (Figure 2A). This divergence in expression profiles raises the possibility that the two paralogs may contribute differently during the asexual developmental cycle.

Susceptible soybean leaves were inoculated with *P. sojae*, and transcript levels were monitored at 0, 3, 6, 12, 24, 48, and 72 h post-inoculation (hpi) to examine expression dynamics during host infection [40]. *PsSTT3A* transcript levels remained significantly elevated throughout the experiment (Figure 2B). *PsSTT3B* transcript levels increased significantly during early infection stages (3–24 hpi), peaking at 6 hpi, before declining at later time points, relative to 0 hpi (Figure 2B).

### 3.3. Targeted Disruption of PsSTT3B via CRISPR/Cas9-Mediated Gene Replacement

**Figure 3:**
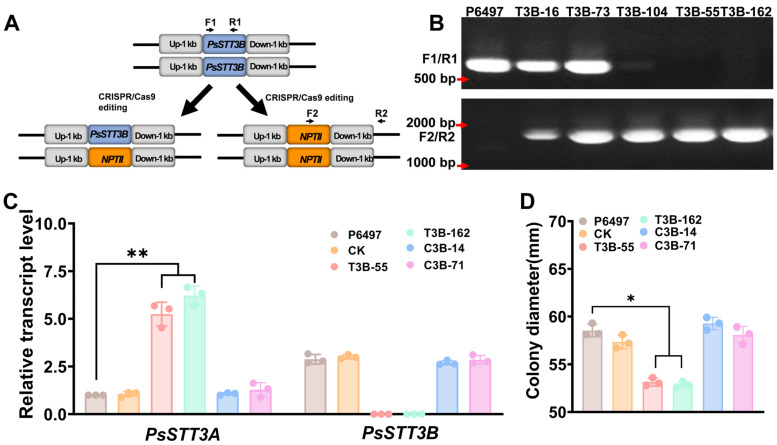
Knockout and verification of *PsSTT3B* in *P. sojae*: (**A**) Diagram showing HDR-mediated replacement of *PsSTT3B* with *GFP*. Blue and orange boxes represent the *PsSTT3B* and *GFP* genes, respectively. (**B**) PCR-based verification of three *PsSTT3B* deletion transformants. Primer pairs F1/R1 and F2/R2 were used to amplify *PsSTT3B* and *GFP*, respectively. (**C**) Transcript levels of *PsSTT3A* and *PsSTT3B* in different *P. sojae* strains were analyzed using RT-qPCR. (**D**) Colony diameters of different *P. sojae* strains. Data represent the mean ± SD of three biological replicates. Asterisks indicate significant deviations from the WT strain P6497 (*, *p* < 0.05; **, *p* < 0.01).

To investigate the biological function of *PsSTT3B*, a CRISPR/Cas9-mediated homologous recombination strategy was employed to replace the *PsSTT3B* coding region with a neomycin phosphotransferase II (*GFP*) resistance cassette in the wild-type strain P6497 [32,33] (Figure 3A). Following G418 selection, two independent deletion mutants (T3B-55 and T3B-162) were obtained. Control transformants (CK) that underwent transformation but retained the native PsSTT3B locus were also isolated (Figure 3B). PCR analysis, together with Sanger sequencing of the amplified fragments, confirmed the integration of the *GFP* cassette and the deletion of the native *PsSTT3B* locus in the knockout strains. (Figure 3B). RT-qPCR further verified that *PsSTT3B* transcripts were undetectable in these mutants, confirming complete gene disruption (Figure 3C).

To determine whether the observed phenotypes were specifically attributable to *PsSTT3B* deletion, in situ complementation was performed by reintroducing the *PsSTT3B* coding sequence into the knockout background [34]. Genetically complemented strains (C3B-14 and C3B-71) restored *PsSTT3B* transcript levels to levels comparable to those observed in the wild type. Interestingly, *PsSTT3A* transcript levels were significantly elevated in *PsSTT3B* deletion mutants during vegetative growth compared with the wild type (Figure 3C), suggesting possible compensatory transcriptional regulation between the two paralogs. However, this upregulation was insufficient to restore wild-type phenotypes.

### 3.4. PsSTT3B Is Required for Vegetative Growth and Asexual Development of P. sojae

Colony growth assays on 10% *v*/*v* V8 agar revealed that *PsSTT3B* deletion notably affected vegetative growth. After 5 days of incubation, *PsSTT3B* deletion mutants exhibited significantly reduced colony diameters compared with the wild-type strain P6497. The radial growth rates of T3B-55 and T3B-162 were reduced by ~12%, indicating impaired hyphal extension (Figure 3D; Supplementary Figure S3). Genetically complemented strains restored colony expansion to levels indistinguishable from those of the wild type, indicating that the growth defect was specifically caused by *PsSTT3B* disruption rather than transformation-associated effects.

**Figure 4:**
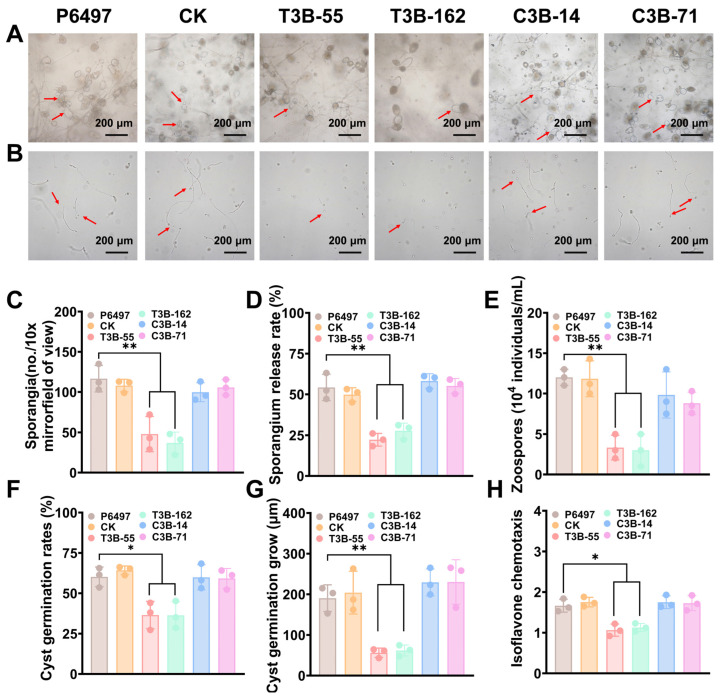
Effects of *PsSTT3B* on asexual development of *P. sojae*: (**A**) Microscopic observation of sporangia and sporangial release in P6497, CK, *PsSTT3B* deletion mutants, and complemented strains at 10× magnification. Red arrows in (**A**) indicate released sporangia. (**B**) Microscopic observation of cyst germination of P6497, CK, the *PsSTT3B*-knockout transformant, and the complemented transformant. Red arrows in (**B**) indicate germinated zoospores. (**C**) Quantification of sporangia production of P6497, CK, the *PsSTT3B*-knockout transformant, and the complemented transformant at 10× magnification. (**D**) Sporangial release rate of P6497, CK, the *PsSTT3B*-knockout transformant, and the complemented transformant. Sporangial release rate = number of released sporangia/(number of released sporangia + number of unreleased sporangia) × 100%. (**E**) Zoospore production of P6497, CK, the *PsSTT3B*-knockout transformant, and the complemented transformant. (**F**) Cyst germination rate of P6497, CK, the *PsSTT3B*-knockout transformant, and the complemented transformant. (**G**) Cyst germ tube length of P6497, CK, the *PsSTT3B*-knockout transformant, and the complemented transformant. (**H**) Isoflavone(ISO)-induced chemotactic response of P6497, CK, the *PsSTT3B*-knockout transformant, and the complemented transformant. The chemotaxis index was calculated as the number of zoospores under isoflavone induction divided by the number under the water control. Sporangium and zoospore production were quantified by examining five microscopic fields per sample, with three independent biological replicates. For spore germination assays, at least 100 spores were assessed per replicate, with three independent biological replicates. Chemotaxis assays were performed using three independent biological replicates, with three microscopic fields per replicate. Data are presented as the mean ± SD of three biological replicates. Asterisks indicate significant differences from the wild-type strain P6497 (*, *p* < 0.05; **, *p* < 0.01).

To determine whether *PsSTT3B* affects asexual reproduction, sporangia formation was quantified under sporulation-inducing conditions. *PsSTT3B* deletion mutants produced significantly fewer sporangia than the wild-type and control strains. Both independent knockout lines indicated an ~54% reduction in sporangial density (Figure 4A,C). Consistent with these results, zoospore release was markedly reduced in the mutants (Figure 4A,D). The number of released zoospores per plate was reduced by >60% relative to the wild type (Figure 4E; Supplementary Figure S4). Complementation fully restored both sporangia production and zoospore release.

Analysis of the expression patterns of *PsSTT3B* and *PsSTT3A* at different developmental stages showed that *PsSTT3A* transcript levels were nearly undetectable in zoospores. Given the normal expression of *PsSTT3B* during the zoospore stage, we evaluated whether it affects zoospore functionality. Germination assays revealed that zoospores derived from *PsSTT3B* deletion mutants exhibited significantly reduced germination rates compared with those derived from the wild type (Figure 4B,F) and produced shorter germ tubes, indicating compromised polarized growth during post-encystment development (Figure 4B,G). Genetically complemented strains showed both germination rate and germ tube length restored to wild-type levels. These data show that *PsSTT3B* influences spore production and the functional competence of zoospores during early developmental transitions.

Host-derived isoflavones act as chemoattractants, guiding zoospores toward soybean roots. Chemotaxis assays using soybean isoflavone gradients as attractant cues showed that wild-type zoospores moved robustly toward isoflavone sources. By contrast, *PsSTT3B* deletion mutants showed significantly impaired chemotactic responses, with reduced accumulation near the attractant region (Supplementary Figure S5), as evidenced by a decrease in the chemotactic index compared with the wild type (Figure 4H). Genetically complemented strains restored chemotactic behavior to wild-type levels (Figure 4H; Supplementary Figure S5). These results indicate that deletion of *PsSTT3B* impairs chemotactic responses in zoospores. However, the underlying basis of this phenotype remains unclear and may reflect defects in signal perception, intracellular signaling, or overall zoospore fitness.

### 3.5. PsSTT3B Is Required for P. sojae Virulence

**Figure 5:**
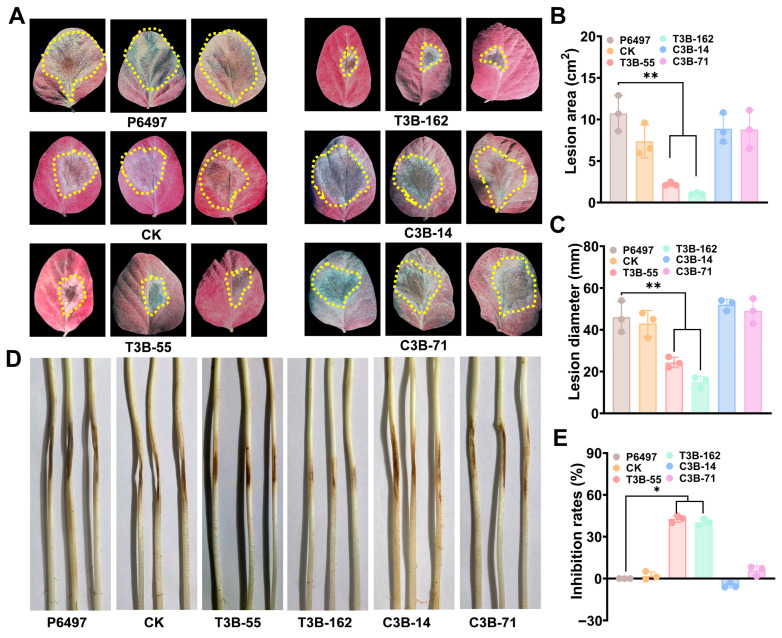
Effects of *PsSTT3B* on the virulence of *P. sojae*: (**A**) Representative lesion symptoms on soybean leaves at 3 dpi. The area within the yellow dashed line represents the diseased region. (**B**) Quantification of diseased leaf area on soybean leaves inoculated with P6497, CK, *PsSTT3B* knockout, and complemented transformants using Image J. Pathogenicity assays were conducted using ten detached soybean leaves. (**C**) Representative lesion symptoms on etiolated soybean seedlings. (**D**) Quantification of lesion diameter on etiolated soybean seedlings inoculated with P6497, CK, PsSTT3B knockout, and complemented transformants using ImageJ. Pathogenicity assays were conducted using six etiolated seedlings per treatment. (**E**) Growth inhibition rate on cellophane relative to P6497. All experiments were performed with three independent biological replicates. Data are presented as the mean ± SD of three biological replicates. Asterisks indicate significant differences from the wild-type P6497 strain (*, *p* < 0.05; **, *p* < 0.01).

Infection assays were conducted on detached soybean leaves and etiolated seedlings to evaluate the biological relevance of these developmental defects. *PsSTT3B* deletion mutants produced significantly smaller lesions compared with the wild-type strain. Lesion areas were reduced by ~20%, indicating compromised virulence (Figure 5A,B). Mutant strains caused delayed disease progression and reduced tissue maceration on etiolated soybean seedlings (Figure 5C,D). Genetically complemented strains restored lesion size and disease severity to levels comparable to those observed in the wild type (Figure 5C,D). The reduced virulence of *PsSTT3B* deletion mutants is consistent with defects in vegetative growth, sporangia production, zoospore production, and zoospore release.

We further examined the growth phenotype of *PsSTT3B* deletion mutants on cellophane. P6497, CK, T3B-55, T3B-162, C3B-14, and C3B-71 were cultured on 10% V8 agar plates lined with cellophane to assess growth on cellophane. Mutants T3B-55 and T3B-162 exhibited significantly reduced mycelial growth on cellophane compared with P6497 and CK (Figure 5E; Supplementary Figure S6). Genetic complementation restored vegetative growth to wild-type levels. These results indicate that deletion of *PsSTT3B* affects mycelial growth on cellophane. As growth on cellophane is commonly used as an indirect proxy for surface-associated growth, these findings suggest that *PsSTT3B* may be involved in processes related to host surface interaction. However, this assay does not provide direct evidence of host tissue penetration in planta.

### 3.6. PsSTT3B Is Required for ER Stress Tolerance

**Figure 6:**
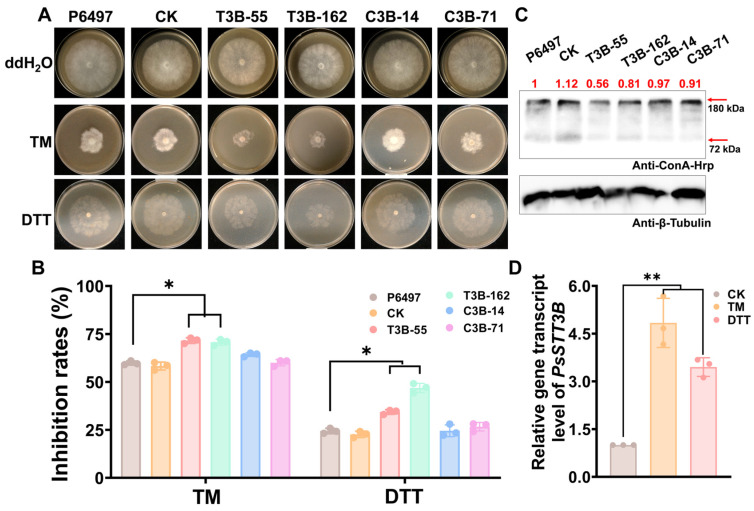
Roles of *PsSTT3B* in ER stress and *N*-glycosylation inhibition in *P. sojae*: (**A**) Mycelial growth assay of all strains on V8 agar supplemented with 0.5 µg mL^−1^ TM or 6 mM DTT. TM, tunicamycin. DTT, dithiothreitol. (**B**) Mycelial growth inhibition rates under TM or DTT treatment. Colony diameters were measured 5 days after treatment. (**C**) Western blot analysis of ConA-binding glycoprotein signals in total mycelial proteins. Total mycelial proteins were extracted and analyzed by Western blotting using an anti-ConA-HRP antibody. Red arrows indicate ConA-reactive bands with slightly reduced intensities in PsSTT3B deletion transformants relative to the wild-type P6497 strain. β-tubulin was used as a loading control. (**D**) Relative *PsSTT3B* transcript levels under TM or DTT treatment. The value of the untreated control (CK) was set to 1 as a reference. All experiments were performed with three independent biological replicates. Data are presented as the mean ± SD of three biological replicates. Asterisks indicate significant differences from the wild-type P6497 strain (*, *p* < 0.05; **, *p* < 0.01).

*PsSTT3B* deletion mutants displayed significantly greater growth inhibition than wild-type and complemented strains when exposed to tunicamycin (TM), an inhibitor of *N*-glycosylation, with the inhibition rate increasing by ~12.3% relative to the wild type (Figure 6A,B). Significant differences were also observed among strains under dithiothreitol (DTT) treatment, which induces ER stress by disrupting disulfide bond formation (Figure 6A,B). Furthermore, *PsSTT3B* transcript levels were strongly induced following TM or DTT exposure (Figure 6D). These results suggest that *PsSTT3B* may contribute to the maintenance of endoplasmic reticulum (ER) homeostasis and protein folding capacity under stress conditions.

### 3.7. Deletion of PsSTT3B Modifies the Profile of ConA-Binding Glycoproteins in Mycelium

Because PsSTT3B is a highly conserved catalytic subunit of the OST complex, we sought to determine whether its deletion affects glycoprotein composition in *P. sojae*. Concanavalin A (ConA), a lectin that specifically recognizes oligomannosidic glycans, exhibits high affinity for terminal α-D-mannose and α-D-glucose residues of glycoproteins [41]. ConA-binding assays revealed that several mannosylated protein bands were slightly reduced in *PsSTT3B* deletion mutants compared with the wild-type P6497 strain (Figure 6C). These results suggest that loss of PsSTT3B may impair the accumulation of high-mannose-type *N*-glycans on a subset of proteins in *P. sojae*. Because specific PsSTT3B-dependent glycoprotein substrates were not identified, these data should be interpreted as supportive biochemical evidence rather than definitive mechanistic proof.

## 4. Discussion

### 4.1. PsSTT3B-Mediated N-Glycosylation Contributes to Development and Pathogenicity

*N*-glycosylation is a fundamental post-translational modification in eukaryotes that shapes protein folding, stability, quality control, trafficking, and secretion [4,42]. Because many secreted and membrane proteins rely on *N*-glycans to attain native conformations and reach their proper destinations, perturbation of *N*-glycosylation can cause pleiotropic defects in development and host–microbe interactions [16]. The OST complex catalyzes the transfer of a preassembled oligosaccharide from a dolichol-linked donor to nascent polypeptides in the ER, and STT3 constitutes the catalytic core of the complex [5]. Although the function of OST has been well characterized in yeast, mammals, and plants, the mechanistic contributions of STT3 paralogs to oomycete development and virulence remain less well defined [25]. Here, we provide genetic and phenotypic evidence that PsSTT3B is a key determinant of *P. sojae* fitness, contributing to vegetative growth, asexual reproduction, zoospore performance, host-directed chemotaxis, pathogenicity, and tolerance to *N*-glycosylation inhibition. Importantly, ConA-based lectin blot analysis provided supportive biochemical evidence for altered high-mannose-type glycoprotein profiles in *PsSTT3B* deletion mutants, although the specific PsSTT3B-dependent glycoprotein substrates remain unidentified.

### 4.2. Possible Non-Equivalent Roles of STT3 Paralogs in Asexual Development

A notable feature of oomycete genomes is the frequent presence of two STT3 paralogs, typically denoted STT3A and STT3B [25]. Distinct STT3 isoforms differ in OST composition and glycosylation timing: STT3A favors co-translational glycosylation, whereas STT3B contributes to post-translational glycosylation and “missed sequon” completion under certain conditions [5,43]. Whether an analogous division of labor applies to oomycetes remains unresolved. However, our phylogenetic and expression data, together with mutant phenotypes, suggest that *PsSTT3A* and *PsSTT3B* may not be fully functionally redundant. *PsSTT3B* is broadly expressed across developmental stages and induced during early infection, whereas *PsSTT3A* is strongly downregulated in zoospores. Moreover, *PsSTT3A* transcript levels increase in *PsSTT3B* knockout strains, consistent with a compensatory transcriptional response; however, this upregulation is insufficient to restore wild-type phenotypes. These patterns are consistent with the possibility that the two paralogs may be regulated differently and may contribute unequally across developmental contexts, with *PsSTT3B* appearing to make a comparatively stronger contribution during zoospore-associated transitions and early infection in the present dataset. However, these conclusions are based on indirect evidence, and direct biochemical or substrate-level analyses would be required to determine whether true functional specialization exists between *PsSTT3A* and *PsSTT3B*.

Loss of *PsSTT3B* caused a pronounced reduction in sporangia production and zoospore release, indicating that efficient asexual reproduction in *P. sojae* depends on intact *N*-glycosylation capacity. Sporulation and zoospore differentiation are expected to impose high demands on ER proteostasis and secretory throughput, as they require membrane remodeling, cell wall biogenesis, and the coordinated deployment of secreted enzymes and structural components [4,44]. Thus, the observed sporulation phenotypes are consistent with a model in which *PsSTT3B*-dependent glycosylation supports the maturation and trafficking of one or more glycoprotein modules required for sporangia and zoospore production. *PsSTT3A* may also contribute to these processes, although whether its contribution differs from that of *PsSTT3B* remains to be clarified experimentally [26].

### 4.3. PsSTT3B Is Associated with Zoospore, Early Infection Processes, and ER Homeostasis

A major insight from this study is that *PsSTT3B* influences not only propagule production but also the functional competence of zoospores, highlighting the zoospore stage as a critical window for *PsSTT3B* action. *PsSTT3B* knockout strains produced zoospores with reduced germination rates and shorter germ tubes, indicating impaired post-encystment development and polarized growth, in contrast to *PsSTT3A* [26]. Establishment of polarity and sustained germ tube elongation depend on coordinated vesicle trafficking, membrane expansion, and localized cell wall deposition—processes that are particularly sensitive to defects in secretory and membrane-associated proteins [45]. In parallel, *PsSTT3B* deletion mutants exhibited reduced isoflavone-induced chemotaxis, manifested as diminished accumulation near attractant cues and a lower chemotactic index. Because host-derived isoflavones can guide *Phytophthora* zoospores toward soybean tissues, reduced chemotaxis provides a plausible phenotypic link between *PsSTT3B* function and early infection success. Fewer zoospores, reduced germination competency, weakened polarized growth, and impaired host-directed movement collectively create multiple bottlenecks that can lower the probability of successful penetration and colonization [10,46,47]. Growth on cellophane is an indirect assay widely used as a proxy for surface-associated growth and related host surface interaction processes. However, this method does not fully recapitulate the complex physical and biochemical environment encountered during host infection. Therefore, the reduced growth observed on cellophane in *PsSTT3B* deletion mutants should be interpreted with caution, and additional in planta assays will be required to directly assess penetration ability.

Many receptor-like proteins, transporters, and channels require *N*-glycans for proper folding, stability, and surface accumulation; defects in glycosylation can attenuate ligand perception and downstream signaling [4,16,41]. However, whether such mechanisms apply to *PsSTT3B*-mediated chemotaxis in *P. sojae* remains to be determined. Our results suggest that *PsSTT3B* contributes to processes required for efficient chemotaxis and early developmental transitions but do not yet resolve the underlying molecular basis of the chemotaxis defect. Moreover, increasing evidence indicates that *N*-glycosylation can directly affect the stability and virulence contribution of oomycete secreted proteins. For example, *N*-glycosylation of the apoplastic effector *PsXEG1* protects it from host proteolysis and contributes to virulence [48]. These observations motivate future efforts to identify *PsSTT3B*-dependent glycoproteins in zoospores and during early infection, including potential receptors, signaling components, and secreted virulence factors.

In addition to client proteins, *PsSTT3B* itself contains multiple predicted *N*-glycosylation sites (N128, N526, N570, N577, N581, and N660), including residues conserved with *PsSTT3A*. Previous studies have shown that mutation of a conserved *N*-glycosylation site in *PsSTT3A* (N593) compromises protein stability and function, suggesting a functional role of STT3 glycosylation in oomycetes. Although the glycosylation status of *PsSTT3B* was not examined here, the presence of these conserved sites raises the possibility that it may also be glycosylated, potentially contributing to protein stability or ER function. However, direct biochemical evidence is required to substantiate this hypothesis. Future studies combining site-directed mutagenesis of predicted glycosylation sites with biochemical assays (e.g., PNGase F sensitivity or glycoproteomic analysis) will be required to clarify whether PsSTT3B is glycosylated and to determine its functional significance.

Consistent with a glycosylation-centric role, *PsSTT3B* deletion increased sensitivity to TM, and *PsSTT3B* transcript levels were induced upon exposure to TM [49]. TM blocks the committed step of the dolichol-linked oligosaccharide pathway and elicits ER stress by promoting the accumulation of misfolded glycoproteins [4,41]. These findings suggest that *PsSTT3B* is involved in maintaining ER homeostasis under stress conditions, likely through its role in protein folding and glycosylation. Meanwhile, stress induced by DTT, which disrupts disulfide bond formation, produced weaker or distinct strain-specific effects in our assays, suggesting that *PsSTT3B* function may be closely linked to *N*-glycosylation and general ER stress. However, these observations provide only indirect physiological evidence, and direct assessment of unfolded protein response activation is required to establish a mechanistic link.

### 4.4. Comparison with Previous Studies

Our findings are consistent with previous work on related *Phytophthora* spp. In *P. capsici*, disruption of *PcSTT3B* reduces growth and virulence, increases sensitivity to TM, and elicits compensatory changes in the STT3 paralog [25]. The concordance between *PcSTT3B* and *PsSTT3B* phenotypes supports a conserved role for STT3B-type OST activity in *Phytophthora* [25]. Unlike *PcSTT3B*, the pronounced zoospore-stage phenotypes observed here—including zoospore germination, polar growth, and isoflavone-induced chemotaxis—raise the possibility of species-specific functional differences. Species-specific ecological adaptation may therefore determine which glycoprotein-dependent processes are most vulnerable in each pathogen [46,47,50].

### 4.5. Limitations and Future Directions

From a disease management perspective, our results highlight *N*-glycosylation as a potential intervention point, as perturbation of this pathway may simultaneously reduce propagule output and impair host targeting and early infection events. In oomycete epidemics, asexual reproduction and zoospore-mediated host location are central drivers of disease spread [51]. Therefore, interventions that limit sporulation and compromise zoospore fitness could disrupt multiple steps of the disease cycle. However, OST and *N*-glycosylation are highly conserved across eukaryotes, implying that any practical application must emphasize selectivity and safety for non-target organisms [41,52]. This study establishes, at the functional level, that *PsSTT3B* is a key gene involved in *Phytophthora* pathogenesis and development, providing a conceptual basis for targeting the *N*-glycosylation pathway. However, it does not directly support drug development. Further studies, including the identification of subtype-specific inhibitors, toxicity evaluation in non-target organisms, and field safety assessments, will be required to assess its practical applicability.

Several limitations of this study warrant consideration. First, the direct *PsSTT3B*-dependent glycoprotein substrates remain unknown; identifying these substrates—particularly in zoospores and early infection stages—will be critical to linking OST activity to specific developmental and signaling pathways. Comparative glycoproteomics and secretome profiling across wild-type, knockout, and complemented strains are likely to be informative. Second, rigorously testing a division-of-labor model between *PsSTT3A* and *PsSTT3B* will require direct assays of glycosylation efficiency for defined substrates or “skipped sequon” models [43]. Third, integrating ER quality control and unfolded protein response markers with chemical stress assays would clarify whether *PsSTT3B* modulates specific arms of ER proteostasis. Addressing these questions will help refine a mechanistic framework in which *PsSTT3B*-dependent *N*-glycosylation supports glycoprotein maturation required for sporulation, zoospore fitness, host perception, and pathogenic fitness in *P. sojae*.

## 5. Conclusions

In summary, this study identifies and functionally characterizes PsSTT3B as a catalytic subunit of the OST complex in *P. sojae*. Our results demonstrate that *PsSTT3B* is required for normal vegetative growth, asexual development, zoospore performance, and full virulence. Disruption of *PsSTT3B* leads to defects in sporangia and zoospore production, reduced germination capacity, impaired chemotactic response, and attenuated pathogenicity on soybean, accompanied by increased sensitivity to chemical stress affecting ER homeostasis. These findings, together with Western blot results, provide indirect evidence suggesting an association between *PsSTT3B*-mediated *N*-glycosylation and developmental progression and pathogenic fitness, although the underlying mechanisms and specific glycoprotein substrates remain to be determined. In addition, the partial transcriptional compensation of *PsSTT3A* in *PsSTT3B* deletion mutants suggests non-equivalent contributions between the two paralogs, but further biochemical and substrate-level analyses will be required to clarify their distinct roles. From an applied perspective, this study provides a conceptual framework for exploring *N*-glycosylation-related pathways as potential intervention points in oomycete disease management. However, given the high conservation of the OST complex across eukaryotes, any practical application will require careful consideration of target specificity and biosafety. Future studies focusing on substrate identification, glycoproteomic profiling, and selective inhibition strategies will be essential to evaluate the feasibility of translating these findings into disease control approaches. These findings may also provide a basis for exploring gene-targeted disease control strategies in soybean using biotechnological approaches such as host-induced gene silencing or spray-induced gene silencing.

## Data Availability

The original contributions presented in this study are included in the article/Supplementary Material. Further inquiries can be directed to the corresponding author.

## Supplementary Materials

The following supporting information can be downloaded at: https://www.mdpi.com/article/10.3390/jof12040274/s1, Figure S1: Predicted transmembrane topology of PsSTT3B was generated using Protter; Figure S2: Schematic representation of putative *N*-glycosylation sites in PsSTT3B protein; Figure S3: Colony morphology of different *P. sojae* strains cultured on V8 agar for 5 d in the dark; Figure S4: Microscopic observation of zoospores from P6497, CK, *PsSTT3B* deletion mutants, and complemented strains at 10× mirror magnification power; Figure S5: Microscopic observation of isoflavone-induced zoospore chemotaxis in P6497, CK, *PsSTT3B* deletion mutants, and complemented strains at 4× mirror magnification power; Figure S6: Effects of *PsSTT3B* deletion on colony growth on cellophane; Table S1: Primers used in this study.
