# Supplementary material for: The Oligosaccharyltransferase Catalytic Subunit PsSTT3B Is Required for Asexual Development and Pathogenicity in Phytophthora sojae
Source: J Fungi (Basel). 2026 Apr 9;12(4):274. doi: 10.3390/jof12040274 (PMC13117519; doi:10.3390/jof12040274)
Supplement: Supplementary file 1 [file jof-12-00274-s001.zip › jof-4218894-supplementary figures.pdf]

Supplementary figures

Figure S1 Predicted transmembrane topology of PsSTT3B was generated using Protter.
Figure S2 Schematic representation of putative *N*-glycosylation sites in PsSTT3B protein.
Figure S3 Colony morphology of different *P. sojae* strains cultured on V8 agar for 5 d in the dark.
Figure S4 Microscopic observation of zoospores from P6497, CK, *PsSTT3B* knockout mutants, and complemented strains at 10 x mirror magnification power.
Figure S5 Microscopic observation of isoflavone-induced zoospore chemotaxis in P6497, CK, *PsSTT3B* knockout mutants, and complemented strains at 4 x mirror magnification power.
Figure S6 Effects of *PsSTT3B* deletion on colony growth on cellophane.

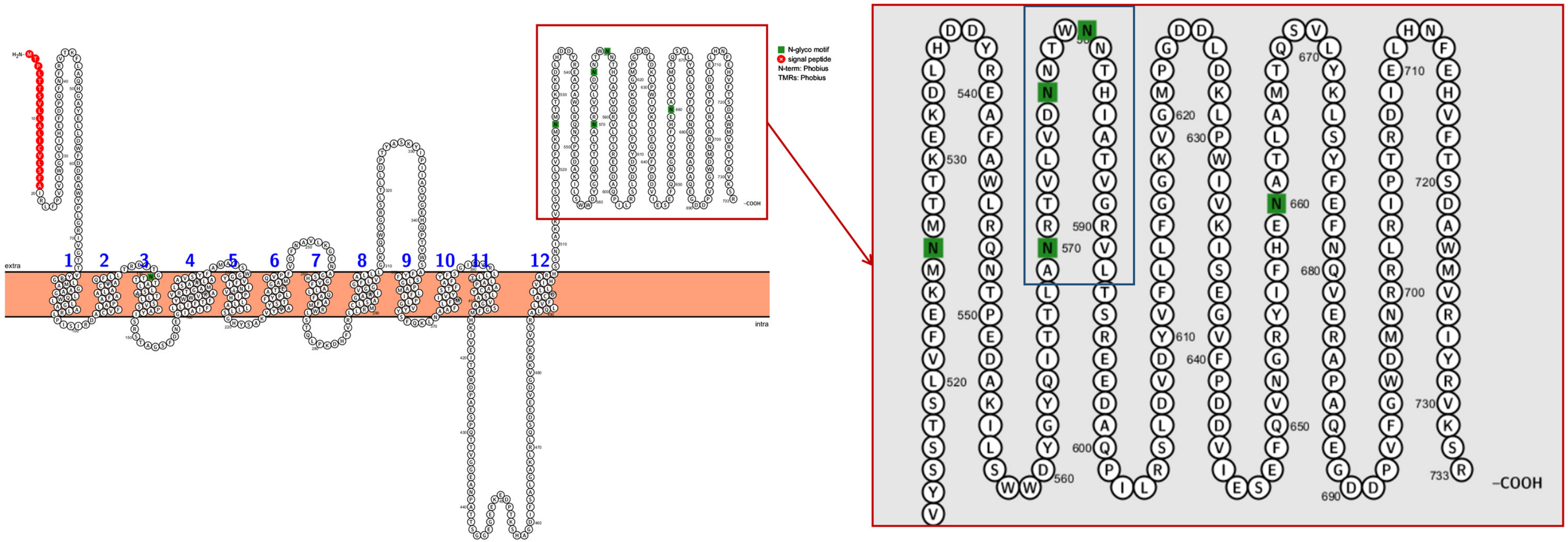

Figure S1 Predicted transmembrane topology of PsSTT3B was generated using Protter.

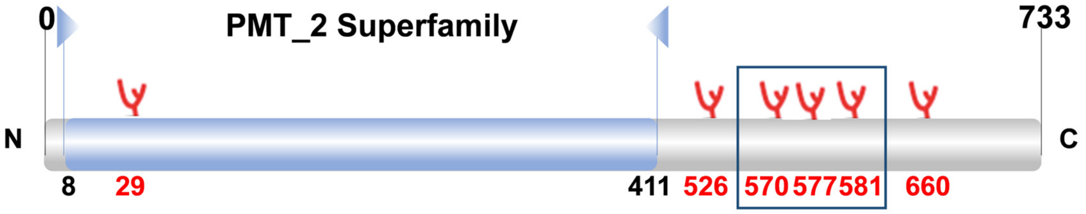

Figure S2 Schematic representation of putative *N*-glycosylation sites in PsSTT3B protein.

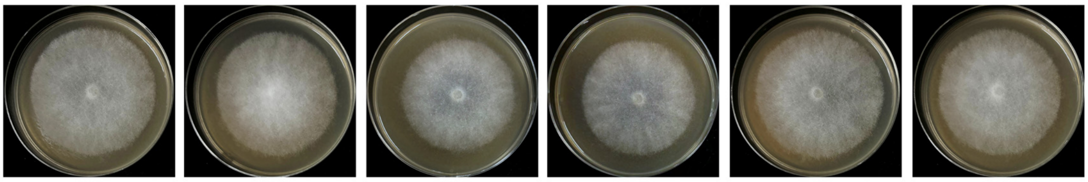

Figure S3 Colony morphology of different *P. sojae* strains cultured on V8 agar for 5 d in the dark. Mycelial growth assays were performed with three independent biological replicates, each including three technical replicates. Colony diameters were measured along two perpendicular axes and averaged.

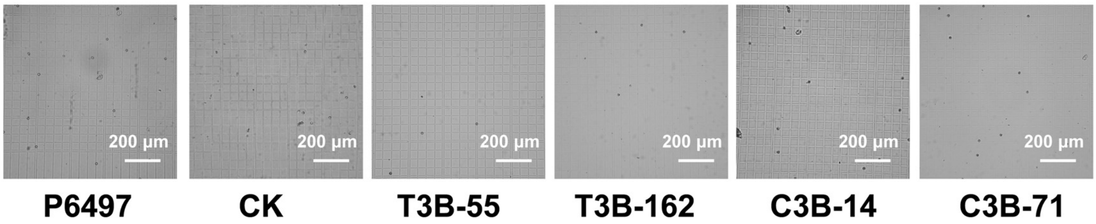

Figure S4 Microscopic observation of zoospores from P6497, CK, *PsSTT3B* knockout mutants, and complemented strains at 10x mirror magnification power.

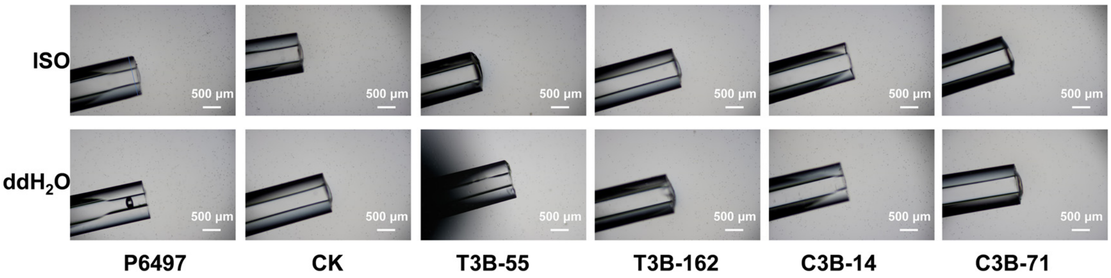

Figure S5 Microscopic observation of isoflavone-induced zoospore chemotaxis in P6497, CK, *PsSTT3B* knockout mutants, and complemented strains at 4 x mirror magnification power.

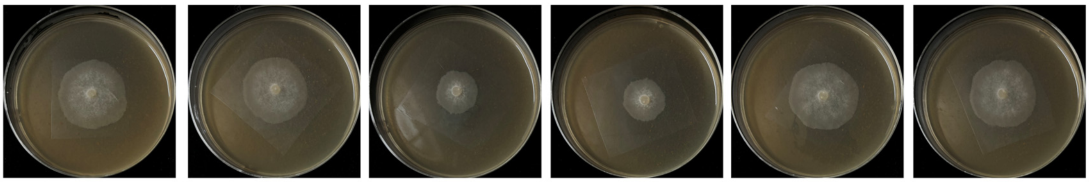

P6497 CK T3B-55 T3B-162 C3B-14 C3B-71

Figure S6 Effects of *PsSTT3B* deletion on colony growth on cellophane. Colony diameters of P6497, CK, PsSTT3B knockout mutants, and complemented strains grown on cellophane-covered plates. Mycelial growth assays were performed with three independent biological replicates, each including three technical replicates. Colony diameters were measured along two perpendicular axes and averaged.
